# Interactive effect of dietary metabolizable energy levels with amino acid density in male broiler chickens: Carcass yield, nutrient intake, digestibility and excretion

**DOI:** 10.1016/j.psj.2024.104530

**Published:** 2024-11-10

**Authors:** Mehdi Toghyani, Shemil MacElline, Peter H. Selle, Sonia Y. Liu

**Affiliations:** a School of Life and Environmental Sciences, Faculty of Science, The University of Sydney, Camperdown NSW 2006, Australia; b Poultry Research Foundation, The University of Sydney, Camden NSW 2570, Australia; c Sydney School of Veterinary Science, The University of Sydney, Camperdown NSW 2006, Australia

**Keywords:** Nutrient density, Nutrient digestibility, Carcass yield, Meat quality

## Abstract

This study aimed to assess the interactive effects of ME and digestible amino acid (AA) densities on carcass yield, breast meat quality, nutrient digestibility, and excretion in male broiler chickens. Twelve experimental diets arranged as a 4 × 3 factorial array with 4 levels of ME (standard, -50, -100, -150 kcal) and 3 digestible AA (standard, +3.0 and +6.0%) were offered to 2400 day-old male Ross 308 broiler chickens. Diets were replicated 8 times with 25 birds per replicate, and fed for starter (0-10 d), grower (11-24 d), finisher (25-35 d), and withdrawal (36-42 d) phases, with consistent reduction of ME and increase in AA density for each phase. On day 24, three birds per replicate were euthanized to collect digesta for nutrient digestibility and excreta samples were collected on day 40 for nutrient excretion analysis. On day 42, four birds per replicate were euthanized for carcass yield and quality evaluation. The birds' feed intake, along with calculated ME and AA levels, were used to determine nutrient intake. No interactions were observed between ME and AA densities on carcass characteristics, nutrient digestibility, and excretion (P > 0.05). Reducing ME density linearly increased breast meat yield and decreased abdominal fat, regardless of AA density (P < 0.01). Increasing AA density enhanced breast meat yield but increased white striping and woody breast scores in a linear manner (P < 0.01). Dietary treatments didn't affect ileal digestibility of crude protein and gross energy, except for improved starch digestibility with 6.0% AA density (P < 0.01). Crude fat digestibility decreased linearly with decreased ME density, while reducing ME density linearly decreased fat, calcium, copper, and manganese excretion (P < 0.01). Increasing AA density linearly increased nitrogen excretion, but reduced calcium and iron excretion (P < 0.05). Over the entire production period lower ME and higher AA density increased digestible lysine intake (mg/bird/day; P < 0.01). In summary, these results suggest that dietary ME and AA density, independently, affect carcass yield, breast meat quality, nutrient digestibility, and excretion.

## Introduction

Poultry meat continues to be the dominant and most affordable animal protein source for human consumption (OECD-FAO, 2023). In the last decades, genetic selection and precise nutrition unequivocally have been identified as the key drivers for the continuous improvement in productive traits of broiler chickens (Aftab, 2019; Fancher, 2014). The major components of efficiency associated with genetic selection have been noted to be increased appetite and a shift in composition of gain as reviewed by Aftab (2019). Recent data from commercial breeders (Aviagen, 2022) predict a breast meat yield of 25.1% at a bodyweight of 2.6 kg, while this figure was forecast at 22.0% at similar bodyweight, back in 2014 (Aviagen 2014), representing an increase of 14% in breast meat yield over a 10-year period. Supporting this trend, Heger et al. (2014) reported a breast meat yield averaging 20.2% of live weight at 35 days in Ross 308 broilers, while more recent findings from Maqsood et al. (2022) indicate an increase to over 24.0% for the same breed and age. However, this rise in breast meat yield has coincided with an increased incidence of breast muscle quality defects, specifically woody breast and white striping. These conditions are believed to be linked to the rapid growth and high breast muscle deposition in modern broilers, which can lead to muscle fiber degeneration and connective tissue abnormalities, ultimately affecting meat quality (Kuttappan et al., 2016). Furthermore, an increased appetite is expected to affect the digestibility of nutrients, particularly ME and crude protein/amino acids (**AA**) (Svihus, 2011). However, no fundamental changes have been noticed in terms of digestibility or metabolizability of diets with recent genetic improvements of broiler chickens (Aftab, 2019).

Beside breeding programs, the key dietary factors fuelling such improvements are dietary ME and AA and the balance between these two nutrients, or in another word, AA to ME ratio which also determines the efficiency of feed utilisation (Barekatain et al., 2021). Simultaneously, both ME and AA relative to other nutrients, represent a large portion of diet cost (Kidd et al., 2004). Thus, nutrient density not only is a key factor affecting the growth rate, carcass yield and quality, and in some instances the overall health of broilers, it also affects the cost effectiveness of broiler chicken production (Brickett et al., 2007; Zhai et al., 2013). Supplying ME and AA in excess of the actual requirements, or an imbalanced AA to ME ratio, may also impact on nutrient excretion to the environment and challenge the sustainability of broiler chickens’ production. Therefore, optimizing dietary ME levels and AA density is crucial for maximizing the productivity, profitability, and sustainability of production.

Being the most limiting and concurrently the most expensive components of broiler chicken diet, measuring the optimal dietary ME and AA, and their ratios have been subjected to numerous studies (Barekatain et al., 2021; Tran et al., 2021; Zhai et al., 2014). The research output over the past two decades concludes that when assessing changes in nutrient needs for modern broilers, the rise in demand for AA is relatively greater compared to that for ME. (Gous, 2010; Maharjan et al., 2021b). Therefore, in late 2022, both major broiler breeder companies revised their recommended specifications for dietary ME and AA density, with ME being reduced by 50 to 100 Kcal and AA increased by over 10% (Aviagen, 2022; Cobb-Vantress, 2022). To satisfy the genetic potential and maximize profitability of newly developed strains of broiler chickens, the dietary ME and AA and their balance in the feed must be continuously evaluated and optimized. Scant information is available exhibiting if these revised specifications could further be adjusted in an attempt to improve breast meat yield and quality and reduce nutrient excretion without impacting other productive traits. Thus, the current feeding trial was designed to determine the interaction between dietary ME levels with AA density against the latest Ross 308 recommendations (Aviagen, 2022) on carcass yield components, breast meat quality, nutrient digestibility and excretion in male broiler chicks reared to 42 days of age.

## Material and methods

### Birds and experimental design

All the experimental protocols and procedures for the present study were reviewed and approved by the University of Sydney Animal Ethic Committee (protocol number AEC2022/2185). A total number of 2400 day-old male Ross 308 chicks were obtained from a commercial hatchery (Goulburn, NSW 2580). Upon arrival, birds were group weighed and assigned into their respective treatments into 96 floor pens. Each treatment was replicated 8 times with 25 birds per replicate. The feeding study consisted of 12 dietary treatments designed as a 4 × 3 factorial arrangement, which included four levels of dietary ME (standard, -50, -100 and -150 kcal/kg) and 3 levels of AA densities (standard, +3.0% and +6.0%) for each phase of the study. The diets were formulated to Ross 308 nutrients specification (Aviagen, 2022) and the reduction of ME and increase in AA density was applied to the base levels recommended by the breeder.

Prior to diet formulation, representative subsamples of wheat, soybean meal, meat and bone meal, canola meal and canola seed were analyzed by near-infrared spectroscopy to predict proximate analysis, digestible amino acid concentrations, and ME using AMINONIR®PROX, AMINONIR®NIR, and AMINONIR® NRG (Evonik Nutrition & Care, Hanua, DE), respectively.

Diets were based on wheat (11.5 % CP; ME 3180 kcal), soybean meal (46.0 % CP; ME 2400 kcal), meat and bone meal (47.0 % CP; ME 2000 kcal), solvent canola meal (37.5 % CP; ME 1980 kcal) and canola seed (21.0 % CP; ME 4500 kcal), without any inorganic phosphate sources. A phytase dose of 2000 FYT/kg (Ronozyme HiPhorius 10) was included across all the diets with a matrix of 0.20 % Ca, 0.18% available P, 0.02% Na, 30 kcal/kg ME uplift and 50% of the manufacturer recommended matrix for AA. The diets were balanced for the essential amino acids including Lys, M+C, Thr, Trp, Arg, Ile and Val by applying the ideal amino acid ratios recommended by breeder (Aviagen, 2022). There was neither a cap nor a minimum set for dietary crude protein. Realtime ingredients pricing were used to formulate the diets and synthetic amino acids including Lys, Met, Thr, Arg, Ile and Val were offered to the diets at market prices.

### Data collection

On day 24, a total of 3 birds per pen were randomly selected, euthanized by intravenous injection of sodium pentobarbitone and the digesta content from distal ileum was collected. Digesta samples from birds within a pen were pooled, homogenized, freeze-dried and ground through 0.5mm screen. Digesta samples were analyzed for the content of the indigestible marker, protein, starch, fat and gross energy.

On day 40 of the trial, 5 birds per pen were randomly selected, placed in tub and fresh excreta samples were collected into 250 ml plastic containers. Fresh excreta were homogeneously mixed and then sub samples were collected into 25 ml containers, freeze-dried and ground through 0.5mm screen. Excreta samples were analyzed for the content of nitrogen, fat, macro and micro minerals.

**Fig. 1:**
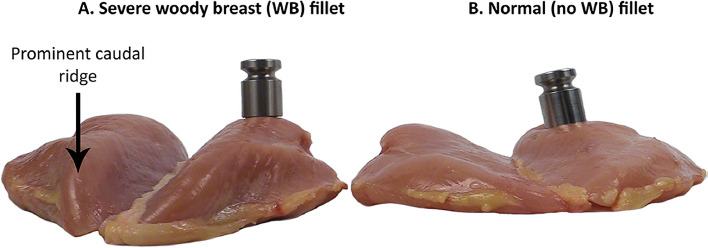
Comparison of severe woody breast (WB) and normal fillets (A and B, respectively). Each fillet has a 200 g weight resting on the cranial portion of the fillet. The severe WB shows no visual signs of compression while the weight on the normal fillet compresses the surface of the fillet. The breast fillets were given a score of 0 = no woody breast, 1 = moderate, 2 = severe and 3 = extreme woody breast conditions. Adopted from Kuttappan et al. (2016).

**Fig. 2:**
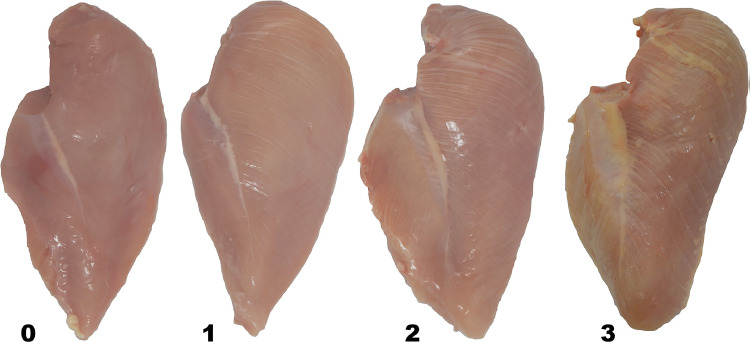
Modified visual scoring scale for white striping in breast fillets where 0 = normal, 1 = moderate, 2 = severe, and 3 = extreme. Normal – No distinct white lines. Moderate – Small white lines, generally < 1 mm thick, but apparently visible on the fillet surface. Severe – Large white lines (1 - 2 mm thick) very visible on the fillet surface. Extreme – Thick white bands (> 2 mm thickness) covering almost entire surface of fillet. Adopted from Kuttappan et al. (2016).

On day 42, a total of 4 birds per pen whose body weight was close to the pen mean were selected and euthanized for carcass analysis. Skinless breast meat (*Pectoralis* major and minor), leg quarter (thigh + drumstick), and abdominal fat pad were removed, weighed, and calculated as g per 1000 g live bodyweight of sacrificed birds. Breast major muscles were also visually examined and scored for the occurrence of woody breast and white striping (Fig. 1, Fig. 2) according to Kuttappan et al. (2016). The frequency of occurrence of each score as a percentage was calculated using the following equation:

$$\frac{\text{Number}\;\text{of}\;\text{observations}\;\text{with}\;a\;\text{specific}\;\text{score}}{\text{Total}\;\text{number}\;\text{of}\;\text{observations}\;}\;\times \;100$$

The average feed intake of birds within a pen for each phase and the entire production period of 0-42 d was used to calculate digestible lysine and ME intake per bird/day using the calculated ME and dLys of each diet.

### Chemical analysis and calculations

Titanium dioxide (TiO_2_) was included in the grower diets at 5 g/kg as an inert marker. Titanium dioxide concentrations were determined in triplicate and duplicate for diets and digesta samples, respectively, by the colorimetric method described by (Short et al., 1996). The ileal digestibility coefficient of nutrients was calculated using the indigestible marker as follows:

$$\text{Ileal}\text{digestibility}\text{coefficient}=1-\left[\text{Ti}O_{2}\text{diet}\left(\%\right)/\text{Ti}O_{2}\text{digesta}\left(\%\right)\right]\times \left[\text{digesta}\text{nutrient}\left(\%\right)/\text{diet}\text{nutrient}\left(\%\right)\right]$$

Duplicate representative composite samples from each diet (as-fed basis) and excreta samples were analyzed for mineral concentration using an inductively coupled plasma-optical emission spectrometer (ICP-OES) (Agilent, Mulgrave, Victoria, Australia) following the methodology described by Maxfield and Mindak (1985). The nitrogen content of raw ingredients, diets, digesta and excreta samples were determined on a 0.25-g sample in a combustion analyzer (FP-2000, LECO Corp., St Joseph, MI, USA) using ethylenediaminetetraacetic acid (EDTA) as a calibration standard, with crude protein being calculated by multiplying percentage nitrogen by a correction factor (6.25) as described by Siriwan et al. (1993).

Starch concentration of the diets and digesta samples were determined by a procedure based on dimethyl sulfoxide, α-amylase and amyloglucosidase, as described in Mahasukhonthachat et al. (2010). The gross energy of diets and digesta were determined by bomb calorimetry using an adiabatic calorimeter (Parr 1281 bomb calorimeter, Parr Instruments Co., Moline, IL). Dry matter and fat content of diets, digesta and excreta were determined using methods of AOAC (2005).

### Statistical analysis

Data were checked for normality and then subjected to statistical analysis using 2-way ANOVA of GLM procedure in JMP®13 (SAS Institute Inc., JMP Software, Cary, NC) to assess the main effects of ME level, AA density, and their interaction. Each pen was considered as an experimental unit and the values presented in the tables are means with pooled standard error of mean (SEM). If a significant effect of treatment was detected, differences between treatments or main effects were separated by least square differences test. Significance was considered at P < 0.05.

Where appropriate, linear regression analysis was performed using the polynomial model analysis in JMP®13 (SAS Institute Inc., JMP Software, Cary, NC) to establish the relationship between dietary ME or AA density with particular variables.

## Results

### Nutrient intake and carcass characteristics

Based on the data presented in Table 1, over the starter period ME intake was only affected by AA density, where birds offered high AA diets had approximately 1.5 kcal per day lower ME intake compared to standard and medium AA density diets (P < 0.05). During the grower and withdrawal phases, increasing AA density increased the ME intake of birds fed standard or -150 kcal ME diets, but had no effect on ME intake of birds offered -50 and -100 Kcal ME diets, resulting in an interaction between ME and AA density (P < 0.05). During the finisher period, decreasing ME by 100 and 150 Kcal decreased ME intake (P < 0.01), while high AA density increased ME intake (P < 0.05). Over the entire production period of 0-42 d, decreasing ME by 150 kcal significantly reduced the average ME intake compared to -50 kcal ME diet, while increasing AA density at both levels increased ME intake (P < 0.01). Reducing ME by 100 or 150 kcal or increasing AA density at both levels increased digestible lysine (dLys) intake of the birds during the starter phase (P < 0.01). An interaction between ME and AA during the grower and withdrawal periods, resulted in reduced dLys intake at standard AA density only when birds received diets with standard ME density (P < 0.05). The dLys intake in the finisher phase increased as the ME of the diet decreased or when AA density increased (P < 0.01). Overall, the average dLys intake (0-42 d) increased when dietary ME decreased, or AA density increased (P < 0.01).

**Table 1:** Broilers ME and digestible Lys intake over different growth periods and the entire production period (0-42 d).

Treatments		ME intake (kcal/bird/day)					Digestible Lys intake (mg/bird/day)				
ME (Kcal)	AA	STR	GRW	FIN	WDRL	0-42 d	STR	GRW	FIN	WDRL	0-42 d
STD	SAA	87.1	304^e^	561	614^d^	371	38.7	118^h^	196	199^f^	133
STD	MAA	88.1	317^a^	563	655^ab^	383	40.3	127cdefg	202	218^cd^	141
STD	HAA	85.3	315abc	559	669^a^	383	40.1	129cde	205	229^ab^	145
-50	SAA	85.9	312abcd	555	635bcd	376	38.8	123^g^	197	209^e^	136
-50	MAA	86.5	311bcd	562	653^ab^	380	40.2	126defg	205	221^bc^	142
-50	HAA	86.5	314abc	575	657^ab^	385	41.4	131^bc^	215	229^ab^	148
-100	SAA	87.8	313abcd	552	633bcd	375	40.3	125^fg^	199	212^de^	139
-100	MAA	87.0	314abc	555	644^bc^	378	41.1	130bcd	205	222^bc^	144
-100	HAA	85.8	316^ab^	558	646abc	380	41.8	134^ab^	212	229^ab^	148
-150	SAA	88.6	308^de^	546	623^cd^	371	41.4	126defg	200	212^de^	139
-150	MAA	87.7	309cde	557	641^bc^	377	42.2	130cde	210	224^bc^	146
-150	HAA	86.3	314abc	551	653^ab^	378	42.8	135^a^	213	235^a^	150
	SEM	0.851	2.17	3.92	5.13	1.95	0.401	0.886	1.43	1.75	0.73
Main effects											
ME											
STD		86.8	312	561^ab^	646	379^ab^	39.7^c^	124	201^b^	216	139^c^
-50		86.3	312	564^a^	648	380^a^	40.1^c^	127	205^a^	220	142^b^
-100		86.9	314	555^bc^	641	378^ab^	41.1^b^	130	205^a^	221	144^ab^
-150		87.5	310	551^c^	639	375^b^	42.1^a^	130	207^a^	224	145^a^
AA											
SAA		87.4^a^	309	554^b^	626	373^b^	39.8^b^	123	198^c^	208	137^c^
MAA		87.3^a^	313	559^ab^	648	380^a^	41.0^a^	128	205^b^	221	143^b^
HAA		86.0^b^	315	561^a^	656	382^a^	41.5^a^	132	211^a^	231	148^a^
Source of variation *P value*											
Energy		0.376	0.232	0.005	0.101	0.009	<.001	<.001	<.001	<.001	<.001
Amino acid		0.036	0.003	0.037	<.001	<.001	<.001	<.001	<.001	<.001	<.001
Energy × Amino acid		0.476	0.024	0.071	0.005	0.247	0.525	0.039	0.059	0.011	0.344

Each value for each treatment represents the mean of 8 replicates of 25 birds each.

a-g Means within a column not sharing a superscript differ significantly at the P < 0.05 level for the treatment effects and at the P level shown for the main effects. ^1^STD: standard ME, SAA: standard amino acid density, MAA: medium amino acid density (+3.0%), HAA: high amino acid density (+6.0%)

Decreasing ME density linearly enhanced breast meat yield (R^2^ = 0.78) and reduced abdominal fat pad weight (R^2^ = 0.96) irrespective of diets AA density (P < 0.01; Table 2). Increasing AA density, at each level of increase, enhanced breast meat yield (R^2^ = 0.97) and decreased abdominal fat pad weight (R^2^ = 0.98; P < 0.01).

**Table 2:** Breast meat, leg quarter and fat pad yield determined on day 42 post hatch.

Treatments		Carcass yield g/kg live BW				
ME (Kcal)	AA	*P*. Major	*P*. Minor	*P*. Total	Leg Qtr.	Fat pad
STD	SAA	195	35.9	231	211	10.05
STD	MAA	201	36.6	238	213	9.71
STD	HAA	206	36.5	243	208	8.73
-50	SAA	200	36.8	237	210	9.92
-50	MAA	202	36.9	239	211	9.42
-50	HAA	209	37.2	247	211	7.78
-100	SAA	209	37.3	246	209	8.61
-100	MAA	210	37.4	248	209	8.23
-100	HAA	213	37.9	251	209	7.74
-150	SAA	206	37.3	244	210	9.09
-150	MAA	209	37.2	246	209	7.69
-150	HAA	212	37.3	249	213	7.27
	SEM	2.16	0.386	2.31	2.25	0.420
Main effects						
ME						
STD		201^b^	36.3^b^	237^b^	211	9.50^a^
-50		204^b^	36.9^ab^	241^b^	211	9.04^ab^
-100		211^a^	37.5^a^	248^a^	209	8.20^bc^
-150		209^a^	37.3^a^	246^a^	211	8.02^c^
AA						
SAA		203^b^	36.8	239^b^	210	9.42^a^
MAA		205^b^	37.0	242^b^	211	8.76^b^
HAA		210^a^	37.2	247^a^	210	7.88^c^
Source of variation *P value*						
Energy		<.001	0.003	<.001	0.764	<.001
Amino acid		<.001	0.411	<.001	0.935	<.001
Energy × Amino acid		0.677	0.913	0.683	0.719	0.558

Each value for each treatment represents the mean of 4 birds per replicate, and 8 replicates per treatment.

a-c Means within a column not sharing a superscript differ significantly at the P < 0.05 level for the treatment effects and at the P level shown for the main effects. ^1^STD: standard ME, SAA: standard amino acid density, MAA: medium amino acid density (+3.0%), HAA: high amino acid density (+6.0%) P: Pect*oral* major and minor muscle

Table 3, Table 4 present data on woody breast and white striping mean scores, along with the percentage distribution across different scores. Dietary ME density did not have any significant effect on woody breast mean scores, however, increasing AA density particularly at high level, significantly increased woody breast mean scores (R^2^ = 0.99; P < 0.01). While the percentages of breast fillets with scores of 0, 1, and 2 remained unaffected by dietary ME and AA densities or their interactions (P > 0.05), a significant ME x AA interaction was observed for percentage of fillets with a score of 3 (P < 0.01). Birds fed high AA density diets within the STD ME density group showed a higher percentage of score 3 fillets compared to those on standard or medium AA densities. Reducing ME density led to a significant linear decrease in white striping mean scores (R² = 0.87) compared to standard ME levels (P < 0.05; Table 4). Conversely, increasing AA density resulted in higher white striping mean scores (R² = 0.97; P < 0.01). Birds fed the STD ME diets showed the lowest percentage of score 2 fillets (P < 0.05) and the highest percentage of score 3 fillets (P = 0.093).

**Table 3:** Woody breast mean scores and the frequency of occurrence across the 4 severity grades in breast fillets determined on day 42 post-hatch.

Treatments		Degree of white stripping				Woody breast mean scores
ME (kcal)	AA	0	1	2	3	
		%	%	%	%	
STD	SAA	0.00	29.17	62.50	8.33^b^	1.79
STD	MAA	0.00	16.67	62.50	20.83^b^	2.04
STD	HAA	0.00	12.50	29.17	58.33^a^	2.46
-50	SAA	0.00	16.67	62.50	20.83^b^	2.04
-50	MAA	0.00	20.83	50.00	29.17^ab^	2.08
-50	HAA	0.00	16.67	70.83	12.50^b^	2.13
-100	SAA	0.00	8.33	75.00	16.67^b^	2.08
-100	MAA	0.00	16.67	62.50	20.83^b^	2.04
-100	HAA	0.00	8.33	54.17	37.50^ab^	2.29
-150	SAA	4.17	25.00	50.00	20.83^b^	1.88
-150	MAA	4.17	12.50	50.00	33.33^ab^	2.13
-150	HAA	0.00	12.50	50.00	37.50^ab^	2.25
	SEM	0.107	0.086	0.088	0.078	0.145
Main effects						
ME						
STD		0.00	19.44	51.39	29.17	2.10
-50		0.00	18.06	61.11	20.83	2.08
-100		0.00	11.11	63.89	25.00	2.14
-150		2.78	16.67	50.00	30.56	2.08
AA						
SAA		1.04	19.79	62.50	36.46	1.95^b^
MAA		1.04	16.67	56.30	26.04	2.07^b^
HAA		0.00	12.50	51.04	16.67	2.28^a^
Source of variation *P value*						
Energy		0.120	0.657	0.141	0.042	0.960
Amino acid		0.608	0.489	0.188	0.026	0.006
Energy x Amino acid		0.807	0.835	0.077	0.010	0.507

Each value for each treatment represents the mean of 4 birds per replicate, and 8 replicates per treatment.

a-c Means within a column not sharing a superscript differ significantly at the P < 0.05 level for the treatment effects and at the P level shown for the main effects. ^1^STD: standard ME, SAA: standard amino acid density, MAA: medium amino acid density (+3.0%), HAA: high amino acid density (+6.0%)

**Table 4:** White striping mean scores and the frequency of occurrence across the 4 severity grades in breast fillets determined on day 42 post-hatch.

Treatments		Degree of white stripping				White striping mean scores
ME (Kcal)	AA	0	1	2	3	
		%	%	%	%	
STD	SAA	8.33	8.33	45.83	37.50	2.29
STD	MAA	0.00	8.33	37.50	54.17	2.58
STD	HAA	0.00	12.50	41.67	45.83	2.67
-50	SAA	0.00	20.83	41.67	37.50	2.17
-50	MAA	4.17	25.00	37.50	33.33	2.00
-50	HAA	1.25	16.67	29.17	41.67	2.12
-100	SAA	8.33	16.67	45.83	29.17	1.79
-100	MAA	0.00	25.00	41.67	33.33	2.08
-100	HAA	0.00	16.67	33.33	50.00	2.33
-150	SAA	4.17	29.17	50.00	16.67	1.71
-150	MAA	12.50	41.67	25.00	20.83	1.83
-150	HAA	8.33	25.00	33.33	33.33	2.08
	SEM	0.055	0.083	0.089	0.107	0.173
Main effects						
ME						
STD		2.78	9.72^b^	41.67	45.83	2.51^a^
-50		5.56	20.83^ab^	36.11	37.50	2.10^b^
-100		2.78	19.44^ab^	40.28	37.50	2.07^b^
-150		8.33	31.94^a^	36.11	23.61	1.88^b^
AA						
SAA		5.21	18.75	45.83	30.21	1.99^b^
MAA		4.17	25.00	35.42	35.42	2.13^ab^
HAA		5.21	17.71	34.38	42.71	2.30^a^
Source of variation *P value*						
Energy		0.547	0.017	0.820	0.093	0.002
Amino acid		0.953	0.409	0.141	0.260	0.043
Energy × Amino acid		0.341	0.942	0.867	0.900	0.696

Each value for each treatment represents the mean of 4 birds per replicate, and 8 replicates per treatment.

a-b Means within a column not sharing a superscript differ significantly at the P level shown for the main effects. ^1^STD: standard ME, SAA: standard amino acid density, MAA: medium amino acid density (+3.0%), HAA: high amino acid density (+6.0%)

### Nutrient digestibility and excretion

As shown in Table 5, dietary treatments had no significant effect on ileal digestibility of crude protein and gross energy (P > 0.05). Starch digestibility was significantly affected by AA density, where increased AA density by 6.0% improved starch digestibility (P < 0.01). Crude fat digestibility was linearly reduced when ME density of the diet decreased (R^2^ = 0.99; P < 0.01). Increasing AA density of the diets, at both medium and high levels, linearly increased nitrogen excretion by more than 14 %, irrespective of ME density (R^2^ = 0.88; P < 0.01). There was no significant effect of dietary AA density or its interaction with dietary ME levels on fat excretion (P > 0.05). However, reducing dietary ME linearly (R^2^ = 0.80; P <.01) decreased fat excretion, where -150 kcal diets reduced fat excretion by close to 50% compared to the standard ME diets (P < 0.05).

**Table 5:** Nitrogen and fat excretion (g/kg DM) determined on day 40, and ileal digestibility coefficient of protein, starch, fat and energy determined on day 24 post hatch.

Treatments		Nutrient excretion		Digestibility coefficient			
ME (Kcal)	AA	N	Fat	CP	Starch	Fat	Energy
STD	SAA	4.72	15.5	0.810	0.972	0.965	0.737
STD	MAA	4.96	17.4	0.805	0.969	0.943	0.720
STD	HAA	5.85	16.4	0.807	0.988	0.953	0.725
-50	SAA	4.95	14.6	0.809	0.961	0.949	0.721
-50	MAA	5.91	15.4	0.792	0.978	0.932	0.717
-50	HAA	5.56	16.5	0.810	0.989	0.946	0.726
-100	SAA	4.95	16.5	0.805	0.979	0.929	0.721
-100	MAA	5.70	16.3	0.791	0.976	0.920	0.709
-100	HAA	5.71	14.6	0.803	0.979	0.934	0.709
-150	SAA	4.57	12.1	0.799	0.973	0.902	0.721
-150	MAA	5.66	10.7	0.811	0.969	0.920	0.725
-150	HAA	5.85	10.1	0.811	0.988	0.919	0.718
	SEM	0.281	1.34	0.008	0.005	0.007	0.007
Main effects							
ME							
STD		5.18	16.4^a^	0.807	0.977	0.954^a^	0.727
-50		5.48	15.5^a^	0.804	0.976	0.943^a^	0.721
-100		5.45	15.1^a^	0.799	0.978	0.928^ab^	0.713
-150		5.36	11.0^b^	0.807	0.977	0.914^b^	0.721
AA							
SAA		4.80^b^	14.6	0.806	0.971^b^	0.936	0.725
MAA		5.56^a^	14.5	0.800	0.973^b^	0.929	0.718
HAA		5.74^a^	14.4	0.808	0.986^a^	0.938	0.720
Source of variation *P value*							
Energy		0.571	<.001	0.677	0.975	<.001	0.099
Amino acid		<.001	0.716	0.418	0.001	0.144	0.328
Energy x Amino acid		0.411	0.388	0.680	0.222	0.127	0.688

Each value for each treatment represents the mean of 3 birds per replicate, and 8 replicates per treatment.

a-c Means within a column not sharing a superscript differ significantly at the P < 0.05 level for the treatment effects and at the P level shown for the main effects. ^1^STD: standard ME, SAA: standard amino acid density, MAA: medium amino acid density (+3.0%), HAA: high amino acid density (+6.0%).

Table 6 summarise the effects of dietary treatments on mineral excretion. Reducing dietary ME linearly decreased Ca (R^2^ = 0.96), Mn (R^2^ = 0.91) and Cu (R^2^ = 0.81) excretion (P < 0.01). Increasing dietary AA levels in the feed linearly decreased Ca (R^2^ = 0.76) and Fe (R^2^ = 0.83) excretion (P < 0.01), but increased K excretion (R^2^ = 0.99; P < 0.001).

**Table 6:** Mineral excretion (g/kg of excreta) measured on day 40 post hatch.

Treatments		Macro minerals g/kg DM				Trace minerals mg/kg DM			
ME (Kcal)	AA	Ca	P	Na	K	Zn	Mn	Cu	Fe
STD	SAA	11.40	9.14	3.86	23.6	257	491	81.3	539
STD	MAA	10.55	9.33	3.91	27.7	237	471	78.6	389
STD	HAA	8.78	8.89	3.89	30.0	222	431	69.8	370
-50	SAA	10.54	9.57	3.63	27.0	227	463	66.1	446
-50	MAA	9.12	9.36	3.88	27.5	219	446	61.1	386
-50	HAA	8.22	9.13	3.93	29.2	230	439	62.2	385
-100	SAA	9.58	9.18	3.60	25.1	224	413	57.5	458
-100	MAA	9.11	9.15	3.37	27.2	216	432	66.0	396
-100	HAA	8.26	9.33	3.81	27.5	212	413	60.3	390
-150	SAA	9.20	9.29	3.66	25.8	223	418	59.9	441
-150	MAA	7.97	9.14	3.96	25.9	218	429	61.4	364
-150	HAA	7.53	9.13	3.63	28.3	216	406	54.8	344
	SEM	0.52	0.38	0.36	1.49	12.5	22.3	3.62	41.5
Main effects									
ME									
STD		10.25^a^	9.12	3.89	27.1	239	465^a^	76.6^a^	433
-50		9.29^ab^	9.35	3.81	27.9	225	449^ab^	63.1^b^	406
-100		8.98^b^	9.22	3.59	26.6	217	420^b^	61.3^b^	415
-150		8.23^b^	9.18	3.75	26.7	219	418^b^	58.7^b^	383
AA									
SAA		10.18^a^	9.29	3.69	25.4^b^	233	446	66.2	470^a^
MAA		9.19^b^	9.24	3.78	27.1^ab^	222	445	66.7	383^b^
HAA		8.20^c^	9.12	3.82	28.8^a^	220	422	61.8	372^b^
Source of variation *P value*									
Energy		0.002	0.896	0.792	0.709	0.152	0.028	<.001	0.530
Amino acid		<.001	0.799	0.882	0.008	0.321	0.244	0.110	0.001
Energy x Amino acid		0.803	0.977	0.971	0.727	0.861	0.865	0.354	0.868

Each value for each treatment represents the mean of 5 birds per replicate, and 8 replicates per treatment.

a-c Means within a column not sharing a superscript differ significantly at the P < 0.05 level for the treatment effects and at the P level shown for the main effects. ^1^STD: standard ME, SAA: standard amino acid density, MAA: medium amino acid density (+3.0%), HAA: high amino acid density (+6.0%).

## Discussion

### Carcass characteristics and breast fillet myopathies

The diet structure and performance parameters of the birds in this feeding study is reported in Toghyani et al. (2024). The nutrient analysis of the experimental diets, which included gross energy, crude protein, total amino acids, starch, and crude fat concentrations, indicated that the desired differences in dietary ME and AA density for each phase were likely achieved.

Reducing dietary energy linearly increased breast meat yield and decreased abdominal fat pad weight. While increasing AA density increased breast meat yield and decreased abdominal fat pat yield. Dietary density of AA, particularly when based on balanced AA ratio, have long been known to play a key physiological role in protein synthesis affecting breast meat yield. Thus, many researchers have reported carcass yield benefits achieved by increasing digestible dietary AA density (Maharjan et al., 2020a; Temim et al., 2000; Vieira and Angel, 2012). Previous studies have also reported lower abdominal fat pad yield in response to increasing dietary AA levels (Mabray and Waldroup, 1981; Maharjan et al., 2020b). Vieira and Angel (2012) stated that the modern high yielding broiler was especially responsive to AA density, particularly lysine. The effect of dietary ME density on fat pad yield is consistent across the literature and in line with our observations, different studies have reported reduced dietary ME resulting in lower fat pad weight (Dozier III and Gehring, 2014; Maharjan et al., 2020b; Majdolhosseini et al., 2019). However, in contrast to current findings, some studies have reported either no effect (Jespersen et al., 2024; Maharjan et al., 2021a; Majdolhosseini et al., 2019) or an increase (Zhai et al., 2014) in breast meat yield by feeding higher ME diets. The higher breast meat yield in response to lower dietary ME observed in this study could be an indirect effect of ME density on FI and consequently AA intake; since the average calculated digestible Lys intake (0-42 d) of 144 and 145 mg/bird/day in birds fed diets with 100 and 150 kcal lower ME, respectively, were statistically higher than 139 mg/bird/day in birds fed standard ME diets. Furthermore, there were statistically significant linear relationship between dLys intake and breast meat yield (R^2^ = 0.69) and abdominal fat pad (R^2^ = 0.81). Brito et al. (2017) suggest that increased digestible Lys intake, within the ‘ideal protein’ concept, promote messenger RNA expression of some genes coded in the mitochondrial electron transport chain (ND1, CYTB, COX I, COX II and COX III), which increases the mitochondrial energy, thereby fostering body protein deposition and breast meat yield.

Over the last decade, broiler producers have progressively noticed the occurrence of woody breast (WB) and white striping (WS) in broilers' *pectoralis* major muscles (breast fillet) (Meloche et al., 2018a). Although the macroscopic, microscopic, and meat quality aspects of these myopathies are well understood, the underlying causes are not fully known. Research studies have emphasized genetics as a primary factor influencing WB and WS (Alnahhas et al., 2015; Bailey et al., 2015b). However, both myopathies have been noted to exhibit low heritability and a significant non-genetic influence (Bailey et al., 2015a), highlighting the substantial impact of environmental and nutritional factors on their occurrence. White striping has been linked to accelerated growth rates from high-calorie diets, with severity potentially exacerbated by diets high in fat and low in protein content (Kuttappan et al., 2012). Interestingly, feeding the low ME diets (lower fat) in this study linearly decreased WS scores with the lowest score recorded for the birds fed diets with 150 kcal/kg reduction in dietary ME. While the higher growth rate in birds fed diets with higher AA density, particularly 6.0% level could explain the increased WB and WS incidence in these birds. Breast meat affected by WS reportedly exhibits specific fatty acid profiles characterized by high levels of monounsaturated fatty acids but deficient in eicosapentaenoic and docosahexaenoic acids, whereas normal breasts are rich in saturated and polyunsaturated fatty acids (Kuttappan et al., 2012). Recently, Li et al. (2024) proposed that alterations in energy metabolism (specifically glycolysis and mitochondrial oxidative phosphorylation) and antioxidant enzyme activity triggered by oxidative stress are associated with the severity of WB syndrome. Marchesi et al. (2019) conducted whole transcriptome analysis of the pectoralis major muscle in an attempt to reveal molecular mechanisms involved in with WS and concluded that a potential homogeneous lack of capillary blood supply in the muscle triggers hypoxia, leading to the activation of glycolysis, calcium signalling, and apoptosis-related genes, thereby facilitating tissue damage and the incidence of WS. Similar to our findings, Cruz et al. (2017) reported that increasing the level of digestible Lys improved breast meat yield as well as induced the occurrence and severity of WS and WB scores. Lysine is a critical AA for muscle growth. Decreasing its levels by 15% during mid-phase growth significantly reduces the incidence of WS and WB without affecting performance (Meloche et al., 2018b).

### Nutrient digestibility and excretion

The digestibility coefficients and excretion of different nutrients are influenced by feed intake and dietary nutrient levels (Svihus, 2011; Teeter et al., 1985). In the current study, despite the differences in feed intake of the birds, being higher at lower ME and higher AA density (Toghyani et al., 2024), digestibility of crude protein and gross energy were not affected by nutrient density. Crystalline or synthetic AA contribute to the nitrogen pool of the diets and are reported not to require digestion and are rapidly absorbed (Wu, 2009). However, in our study the sum of synthetic AA (Lys, Met, Thr, Ile and Val) in the grower diets were not hugely different across different treatments, ranging from 7.7 to 8.3 g/kg of the feed. Nonetheless, birds offered higher AA density diet excreted more nitrogen than the birds on standard AA density. This implies that despite almost identical nitrogen utilization efficiency amongst the treatments, the higher nitrogen input in high AA density diets resulted in higher nitrogen output. This could also be a consequent of higher feed intake and growth rate of the birds with higher AA density. These results contradict the findings of Khoddami (Khoddami et al., 2018), where the authors reported that broiler chickens offered high density diets (higher ME and AA) had significantly higher protein digestibility and protein disappearance rates. The authors attributed this increase in protein digestibility to the higher synthetic AA inclusion in high density diets.

Fat digestibility and excretion followed completely different trends to nitrogen, where lower ME diets (lower supplemental fat) decreased fat digestibility despite reduced fat excretion. Studies indicate that the digestibility of fats and oils in poultry diets decreases with higher inclusion levels (Ravindran et al., 2016). In the current study, the differences in dietary fat content of the grower diets were indeed significant; being 5.6% at standard ME and AA, and 2.6% at -150 Kcal ME and standard AA, indicating more than 110% difference in fat content. Pancreatic enzyme activity varies depending on the type and amount of substrate available for hydrolysis, as observed in rats (Snook, 1971) and other species (Corring, 1980). Similarly, broiler chickens may adapt their digestive system with diet nutrient densities. Being fed the low ME/fat diets from day-old, these birds might have acclimated to low fat diets with lower pancreatic lipase secretion. Likewise, Petit et al. (2007) reported that increasing portion of lipids in mice diet resulted in upregulation of various genes involved in the absorption and metabolization of fat and increased proliferative activity in the jejunum. Furthermore, ileal endogenous fat loss in broiler chickens has been reported to be 1,714 mg/kg of DM intake (Tancharoenrat et al., 2014). In pigs, the apparent digestibility of dietary fat generally increases with added dietary fat, and this has been attributed to a shift in the balance between dietary fat intake and endogenous fat losses (Jørgensen et al., 1993; Kil et al., 2010). Higher dietary fat levels dilute the proportion of endogenous fat losses relative to total fat intake, leading to improved apparent digestibility. Similarly, in reduced ME diets with lower fat content, the total fat measured in ileal digesta proportionally compromised of more ileal endogenous fat, hence, to some extent explaining the lower fat digestibility in those diets.

The decrease in calcium excretion with reduced ME density appears to be more related to lower fat inclusion in these diets rather than a direct response to ME density *per se*. High-fat diets have been reported to affect the absorption and utilization of calcium in poultry (Fuhrmann and Kamphues, 2016). Fat can increase the solubility of calcium salts in the intestine, potentially enhancing calcium absorption. However, excessive fat intake might also interfere with calcium absorption by forming insoluble soaps with calcium in the gut (Bandali et al., 2018; Leeson and Atteh, 1995). Furthermore, fat metabolism can influence acid-base balance in the body. High-fat diets might lead to increased production of acidic metabolites, which can stimulate the excretion of calcium by the kidneys in order to maintain pH balance (Dawson-Hughes 2020). Thus, excess dietary fat could potentially strain kidney function in chickens. This may lead to alterations in renal handling of calcium, potentially increasing calcium excretion through urine (Leeson and Summers, 2005). Conversely, the lower calcium and iron excretion and higher starch digestibility observed with higher AA density could be attributed to the increased growth rate and a larger body frame augmenting the requirements of both Ca and Fe in response to a larger body mass. As both Ca and Fe are necessary for normal growth and development in broilers and support proper skeletal and muscular development.

Both manganese and copper are cofactors of a variety of enzymes involved in bone formation, carbohydrate, lipid, and protein metabolism such as pyruvate carboxylase, superoxide dismutase and glycosyl transferase (Murray et al., 2000; Suttle 2010). These enzymes play crucial roles in the utilization of carbohydrates, fats, and proteins for energy production and growth in broiler chickens. The linear decrease in Mn and Cu excretion with lower ME diets suggests altered energy and protein metabolism in response to diet structure, ME availability and source i.e. fat or carbohydrates. Indeed, no other studies have investigated the relationship between mineral excretion and other dietary components rather than trace mineral source in the feed.

## Conclusions

In conclusion, this study underscores the significance of optimizing dietary ME and AA densities in broiler chicken feed, not only for cost-effectiveness and performance but also for their impacts on carcass yield, quality, and nutrient excretion into the environment. Under the condition of the current study, reducing dietary ME in broiler chicken diets could be a viable strategy to enhance breast meat yield and quality while decreasing abdominal fat pad deposition. Additionally, lower ME diets have the potential to lower feed costs and reduce nutrient excretion. Conversely, higher AA density increases breast meat yield and reduces abdominal fat pad weight but may also increase the incidence of white striping and woody breast conditions. These findings indicate that dietary ME and AA density appear to independently affect carcass characteristics, nutrient digestibility, and excretion.

## Author contributions

All four authors contributed towards conceptualization, methodology and completion of this study and have read and approved this manuscript. **Mehdi Toghyani** and **Sonia Yun Liu** conceived and designed the experiment and were the principal investigators of the relevant project. **Mehdi Toghyani** formulated the diets and drafted the initial manuscript. **Shemil Priyan Macelline** and **Mehdi Toghyani** conducted the feeding study and statistical analyses. **Peter Henry Selle** assisted manuscript and data interpretation. All authors contributed to further editing and review of the manuscript. **Mehdi Toghyani** was responsible for the final editing and submission of the manuscript.

## Disclosures

We declare that we have no financial and personal relationships with other people or organizations that can inappropriately influence our work, and there is no professional or other personal interest of any nature or kind in any product, service and/or company that could be construed as influencing the content of this paper.
